# Nonrigid Registration of Prostate Diffusion-Weighted MRI

**DOI:** 10.1155/2017/9296354

**Published:** 2017-06-27

**Authors:** Lei Hao, Yali Huang, Yuehua Gao, Xiaoxi Chen, Peiguang Wang

**Affiliations:** ^1^College of Electronic Information Engineering, Hebei University, Baoding 071000, China; ^2^Key Laboratory of Digital Medical Engineering of Hebei Province, College of Electronic and Information Engineering, Hebei University, Baoding 071000, China; ^3^Renji Hospital, Shanghai Jiaotong University School of Medicine, Shanghai 200127, China

## Abstract

Motion and deformation are common in prostate diffusion-weighted magnetic resonance imaging (DWI) during acquisition. These misalignments lead to errors in estimating an apparent diffusion coefficient (ADC) map fitted with DWI. To address this problem, we propose an image registration algorithm to align the prostate DWI and improve ADC map. First, we apply affine transformation to DWI to correct intraslice motions. Then, nonrigid registration based on free-form deformation (FFD) is used to compensate for intraimage deformations. To evaluate the influence of the proposed algorithm on ADC values, we perform statistical experiments in three schemes: no processing of the DWI, with the affine transform approach, and with FFD. The experimental results show that our proposed algorithm can correct the misalignment of prostate DWI and decrease the artifacts of ROI in the ADC maps. These ADC maps thus obtain sharper contours of lesions, which are helpful for improving the diagnosis and clinical staging of prostate cancer.

## 1. Introduction

Prostate cancer (PCa) is the second most frequently diagnosed cancer in men in western countries [1–4]. The early detection and treatment of PCa can decrease the death rate [5, 6]. The diagnosis of PCa is based on digital rectal examination, prostate specific antigen, transrectal ultrasonography, systematic transrectal biopsy, and diffusion-weighted magnetic resonance imaging (DWI) [7–10]. DWI depends on the microscopic mobility of water molecule diffusion [11–14]. Pathophysiological processes such as cancer are known to have an impact on cell density, which translates into different diffusion properties. Therefore, DWI can be applied in the detection, localization, and staging of PCa [15, 16]. The ADC map calculated from DWI is an important indicator in diagnosing PCa. The ADC proposed by Le Bihan is a noninvasive measure that provides quantitative information on the diffusion of water molecules in biological tissues [17–20], which explains why the ADC can be used to increase the sensitivity and specificity of the detection of PCa together with biopsy [21, 22].

The optimization of the ADC map brings great benefits in guiding targeted biopsy and in localizing and staging PCa, and it provides a roadmap for treatment planning and detecting residual or locally recurrent cancer after treatment [23, 24]. However, spatial misalignment is common in prostate DWI and leads to errors in the estimation of ADC.

Misalignments are particularly prone to occur in DWI because of motion and deformation, which can induce poor image quality and errors in the resulting ADCs. This situation is particularly serious in the case of elderly patients because of their weak respiratory control. Elderly patients cannot tolerate a supine position or remain still for a long time. In addition, the spatial alignment of the acquired DWI is not guaranteed if the pinched prostate deforms with peristalsis of the pelvic organs during the acquisition. The issue of image quality is commonly addressed by acquiring each DWI several times and averaging them. Despite improving the signal-to-noise ratio (SNR) of the resulting ADC map, this technique does not compensate for spatial misalignment.

To the best of our knowledge, there are no correlative studies that address this misalignment in prostate DWI [15, 25–27]. To align the intraslice images and extract a more accurate ADC map, we present a semiautomatic registration algorithm, which enables us to correct the misalignments of DWI resulting from rigid body shifts, irregular distortions, and the patients' movements during the acquisition. The proposed algorithm evaluated 38 regions of interest (ROI) of 19 prostate DWI datasets. The ADC maps with our algorithm were then quantitatively compared with ADC maps without any image processing. The evaluation is based on the computation of similarity metric and reproducibility measures. The results showed that the visual quality of ADC maps can be improved through our proposed image registration algorithm.

## 2. Materials

### 2.1. Subjects

From August 2012 to January 2013, 19 male patients aged 68.96 ± 11.43 (50–87) years underwent conventional MRI and multi-*b* value DWI. It was found that there was a total of 20 PCa lesions and 15 benign prostatic hyperplasia (BPH) lesions. Their average prostate-specific antigen was 47.97 ± 72.98 (2.85–276.00) ng/ml. The mean Gleason score of tumors was 7.43 ± 1.09 (6–9). All patients underwent prostate 3T endorectal coil MRI followed by standard 14-core transrectal ultrasound-guided systematic (TRUS) biopsy. The experiments were conducted with the approval of the ethics committees of the participating institutions.

### 2.2. MR Images

The MR dataset obtained by the 3.0 Tesla (T) MR Scanner (Philips Achieva 3.0T; Philips Extended MR WorkSpace, Eindhoven, The Netherlands) contained T1-weighted imaging, T2-weighted imaging, DWI, and dynamic contrast-enhanced imaging [7]. DWI data were obtained using a multislice 2D echo-planar imaging (EPI) sequence in the transverse orientation. A single shot EPI sequence involved the following parameters: *b* = 0 s/mm^2^ to 1200 s/mm^2^, repetition time (TR) = 2000 ms, echo time (TE) = 68 ms, slice thickness = 3.0 mm, slice gap = 1 mm, flip angle = 90, field of view = 211 × 211 mm^2^, and total effective scan time = 596 s. The size of the acquisition matrix was 160 × 156, and 16 slices were acquired with an in-plane spatial resolution of 1.31 × 1.31 mm^2^, bandwidth 2985 Hz/pixel, and EPI factor 71. DWI sequences and ADC maps were reviewed by two radiologists independently and separately.

Patients with lesions suspicious for cancer on MRI underwent 14 or more core TRUS biopsies by the operator. All biopsies underwent centralized pathologic evaluation by a pathologist.

## 3. Methods

### 3.1. Algorithm Design: Global Transformation and Local Transformation (Steps 1, 2, and 3)

**Figure 1:**
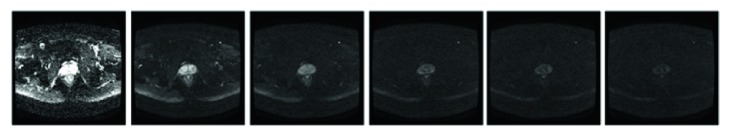
DWI acquired by *b* = 0, 500, 800, 1200, 1500, and 2000 s/mm^2^ shows the tendency of intensity changes.

Figure 1 shows an intraslice DWI sequence with a *b* value of up to 2000 s/mm^2^. Image intensity dramatically reduces with an increase in the *b* value, which leads to prostate boundaries that are fuzzier and more difficult to segment. In addition, the interslice boundaries of the prostate are dynamic and irregular because of the different shooting positions. Thus, feature-based registration is not suitable for prostate DWI. Therefore, an intensity-based registration was applied in our scheme.

**Figure 2:**
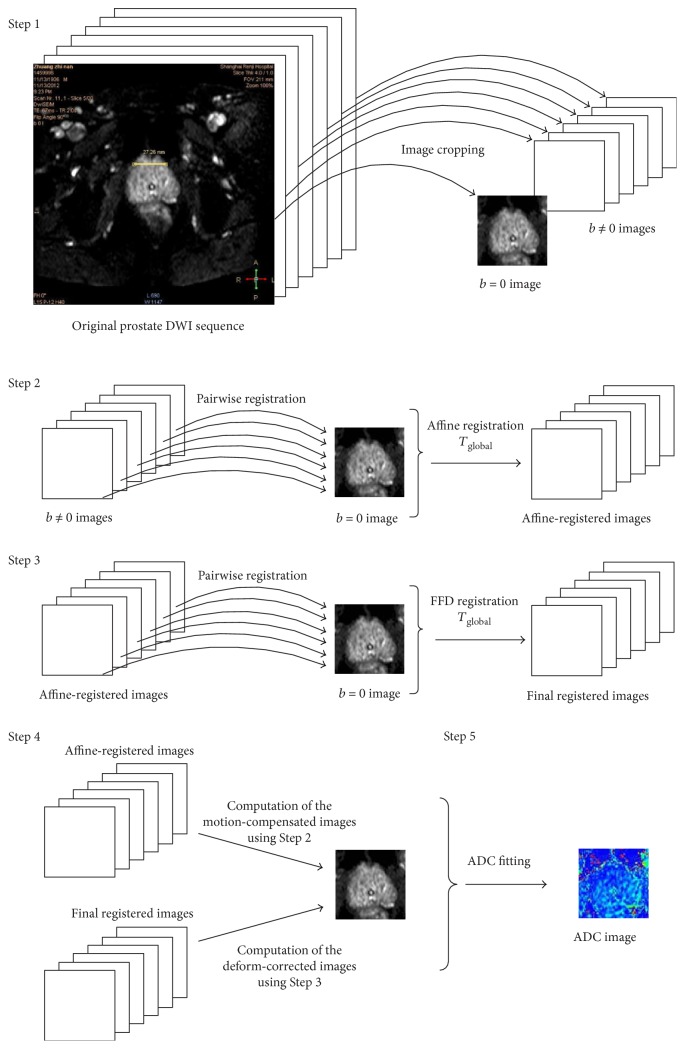
Main steps of the image registration. Step 1: image cropping. Steps 2 and 3: image registration. Step 4: visual and quantitative evaluation of registration. Step 5: ADC fitting.

To correct the motion and deformation of DWI, intensity-based affine and nonrigid image registration algorithms have been developed [28–30]. The various steps are described in this section and illustrated in Figure 2.

**Figure 3:**
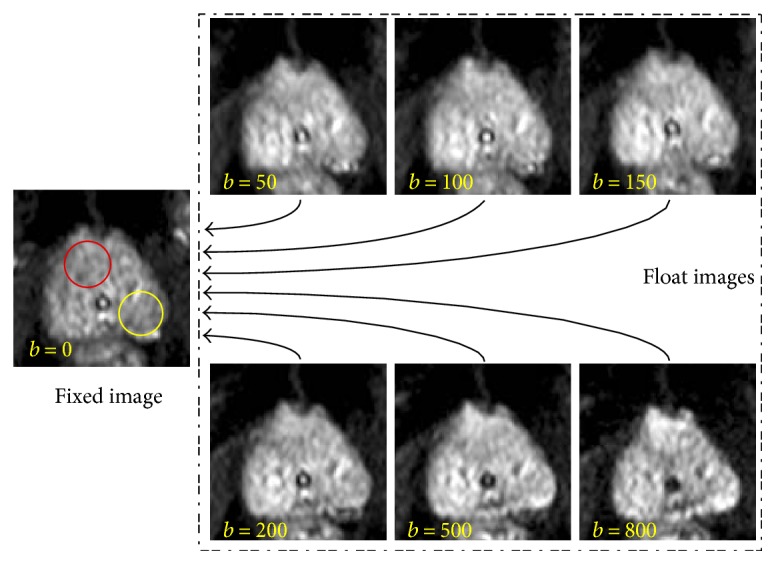
DWI sample acquired with *b* = 0, 50, 100, 150, 200, 500, and 800 in a 61-year-old patient. There was motion and deformation in the region of the prostate. For each patient, two types of elliptic ROIs are manually delineated on *b* = 0 s/mm^2^. The ROI A (red circle) includes the nidus of the prostate, and the ROI B (yellow circle) covers the normal region. The ROIs are propagated differently in the “no processing,” “affine,” and “FFD” schemes.

To improve the accuracy of registration and reduce computing time, the center square region of the prostate is cropped from DWI (intraslice fixed position and uniform size) during image preprocessing (Figure 2, step 1). For the intraslice motion in the DWI sequence, Figure 3 shows the maximum interimage displacement of the prostate: 16 pixels (4.83%) horizontally and 29 pixels (8.9%) vertically. Therefore, we apply affine registration first to compensate for the intraslice motions between the images obtained in step 1, with *b* ∈ {50, 100, 150, 200, 500, 800} s/mm^2^ and *b* = 0 s/mm^2^ (Figure 2, step 2).

The sequences obtained by affine registration are then placed into nonrigid registration (Figure 2, step 3). The original image will be retained if there are unreasonable rotations and scaling in the “affine” scheme. Thus, our algorithm consists of a global rigid transformation and a local nonrigid transformation:

(1) $$\begin{array}{c} T\left(x,y\right)=T_{global}\left(x,y\right)+T_{local}\left(x,y\right). \end{array}$$

The image of *b* = 0 s/mm^2^ in the sequence is chosen as a fixed reference image because it contains more details and has a higher SNR. The principle is to register other images (*b* ≠ 0 s/mm^2^) as float images to the fixed image (Figure 3).

The affine transformation model can describe the overall motion, scaling, and rotation of the prostate DWI obtained under the impact of different imaging times and magnetic field distortion. Therefore, in this study, we apply affine transformation to adjust these spatial misalignments as follows:

(2) $$\begin{array}{c} T_{global}\left(x,y\right)=\left[\begin{array}{cc} s_{x} & 0\\ 0 & s_{y} \end{array}\right]\left[\begin{array}{cc} \cos\theta & \sin\theta \\ -\sin\theta & \cos\theta \end{array}\right]\left[\begin{array}{cc} 1 & k_{y}\\ k_{x} & 1 \end{array}\right]\left[\begin{array}{c} x\\ y \end{array}\right]+\left[\begin{array}{c} ∆x\\ ∆y \end{array}\right], \end{array}$$

where ∆*x* and ∆*y* parameterize translation in the *x* and *y* directions, respectively; *θ* denotes rotation; *s*_*x*_ and *s*_*y*_ denote scaling; and *k*_*x*_ and *k*_*y*_ denote shearing in the *x* and *y* directions, respectively.

Since affine transformation can only roughly correct intraslice misalignment, local and fine transformation should be used to align the irregular intraimage regions of prostate DWI. The local deformation characteristics of the prostate are significantly different among different patients. It is difficult to describe these local deformations via parameterized transformations. Therefore, a free-form deformation (FFD) model based on cubic B-splines is necessary to correct the intra-aligned slices as a highly adaptive tool for modeling soft tissue deformation.

To define a spline-based FFD, we denote the domain of the image area as *Ω* = {(*x*, *y*)∣0 ≤ *x* ≤ *X*, 0 ≤ *y* ≤ *Y*}. A *n*_*x*_ × *n*_*y*_ control mesh denoted by *Φ* is defined and applied to the image with uniform space *δ*. We denote the position of the control points on *Φ* as *Φ*_*i*,*j*_. Then, the FFD can be written as the 2-D tensor product of the familiar 1-D cubic B-splines:

(3) $$\begin{array}{c} T_{local}\left(x,y\right)=\overset{3}{\underset{l=0}{\sum }}\overset{3}{\underset{m=0}{\sum }}B_{l}\left(u\right)B_{m}\left(v\right)\Phi_{i+l,j+m}, \end{array}$$

where *i* = ⌊*x*/*δ*⌋ − 1, *j* = ⌊*y*/*δ*⌋ − 1, *u* = *x*/*δ* − ⌊*x*/*δ*⌋, *v* = *y*/*δ* − ⌊*y*/*δ*⌋, and *B*_*l* _and *B*_*m* _ represent the basic functions of *l*th and *m*th uniform cubic B-splines evaluated at *u* and *v*. They are defined as follows [28]:

(4) $$\begin{array}{c} \begin{array}{l} B_{0}\left(u\right)=\frac{{\left(1-u\right)}^{3}}{6},\\ B_{1}\left(u\right)=\frac{\left(3u^{3}-6u^{2}+4\right)}{6},\\ B_{2}\left(u\right)=\frac{\left(-3u^{3}+3u^{2}+3u+1\right)}{6},\\ B_{3}\left(u\right)=\frac{u^{3}}{6}. \end{array} \end{array}$$

FFD is implemented by manipulating an underlying mesh of control points to correct deformation. A deformation is defined on a sparse, regular grid of control points *Φ*_*i*,*j*_ placed over the float image and is then varied by defining the motion g(*Φ*_*i*,*j*_) of each control point. Using a spline interpolation kernel to compute the deformation values between the control points produces a locally controlled, globally smooth transformation.

The correction effect is affected by the resolution of the control mesh. The higher the resolution is, the finer the deformation correction is. However, since the cost of computation is intolerable, a hierarchal multiresolution that resembles the pyramid approach should be applied.

### 3.2. Optimization

An iterative optimization algorithm is performed with the pyramidal strategy, making this approach a coarse-to-fine strategy [26]. This approach has advantages such as higher convergence radius, more robust performance to local optimums, and faster speed. The motion artifacts are compensated for by a low-resolution strategy in nonrigid registration. Then, the resolution is increased so the local deformation is aligned. The quasi-newton minimization package is applied in local models to reduce the time required to compute the cost function (see (3)) until the termination criteria are satisfied or a maximum number of 800 iterations per resolution is reached [30]. For optimization, the ant colony algorithm minimization method is applied to affine transformation.

### 3.3. Similarity Measure (Step 4)

The most commonly used similarity measures are based on intensity differences, intensity cross-correlation, and information theory, such as normalized cross-correlation (NCC), NMI, and mean-squared error (MSE) [26]. The intensity distribution relationship of DWI is linear. Therefore, NCC is adopted as our cost function while performing the actual registration in this study. The NCC of the fixed image and the float image is defined by

(5) $$\begin{array}{c} NCC\left(I_{1},I_{2}\right)=\frac{1}{N-1}\underset{x}{\sum }\underset{y}{\sum }\frac{\left(I_{1}\left(x,y\right)-\bar{I_{1}}\right)\left(I_{2}\left(x,y\right)-\bar{I_{2}}\right)}{\sigma_{I_{1}}\sigma_{I_{2}}}, \end{array}$$

where *I*_1_ is the fixed image, *I*_2_ is the float image, and *σ*_*I*_1__ and *σ*_*I*_2__ are the standard deviations of *I*_1_ and *I*_2_, respectively. NMI is chosen as a similarity metric to evaluate motion and deformation compensation.

### 3.4. ADC Computation (Step 5)

The ADC values are then extracted by curve fitting from the intraimage registration DWI (see (6)). ADCs are computed from DWI characterized by different diffusion weighting factors (*b* values). Several models to evaluate ADC have been developed: monoexponential model, a biexponential model with two independent fractions, a statistical model with a distribution fraction, and the recent kurtosis model [22]. In this paper, we calculated the ADC with the monoexponential model as follows:

(6) $$\begin{array}{c} S\left(b\right)=S_{0}e^{{-ADC}_{mono}b,} \end{array}$$

where *S*(*b*) is the signal intensity and depends on the strength of diffusion weighting characterized by the *b* factor (*b*). ADC_mono_ is the diffusion coefficient, and *S*_0_ is the signal without applying a diffusion weighting gradient (*b* = 0 s/mm^2^).

## 4. Experiments and Results

### 4.1. Background and Considered Schemes

Nineteen patient datasets were obtained from Renji Hospital. A prostate DWI include 16 sequences, according to different *b* values, and each sequence contained 6–8 images. Each sample was saved in a DICOM format image that contained information on the *b* value and flip angle. A total of 260 transformations were performed, including affine and nonrigid registration.

The effect of registration is evaluated by comparing DWI visualization, NMI, and ADC maps in three schemes (no processing, affine, and FFD). The first scheme is called “no processing,” which means that the ADC curve is obtained with the original sequence. The second scheme is referred to as “affine.” It involves applying translation and zoom to each of the acquired DWIs before obtaining the ADC curve. The third scheme, denoted “FFD,” is that the free-form deformation registration is implemented in the sequence before the ADC curve is fitted.

### 4.2. Visual Evaluation of Image Registration

**Figure 4:**
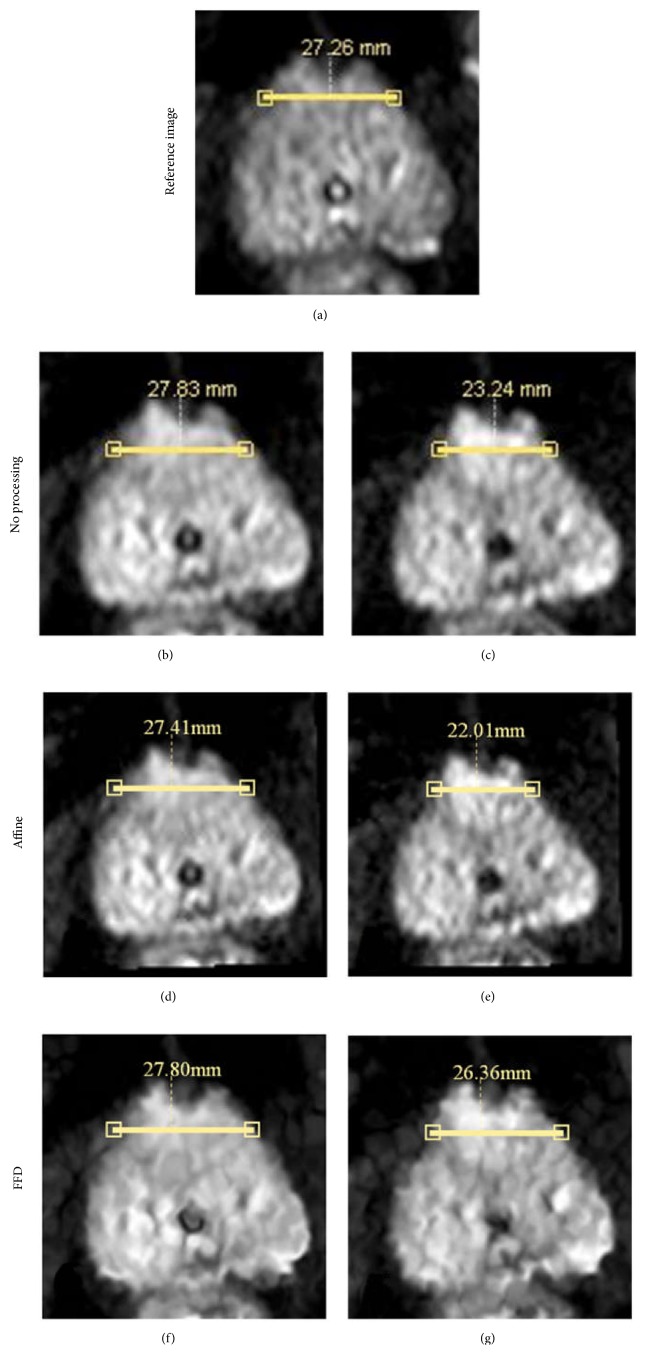
(a) *b* = 0 s/mm^2^ DW image is the reference image with no processing (case 19). (b) and (c) are the respective samples of *b* = 500 s/mm^2^ and *b* = 800 s/mm^2^ DW images in the “no processing” schemes. (d) and (e) are the *b* = 500 s/mm^2^ and *b* = 800 s/mm^2^ DW images in the “affine” schemes. (f) and (g) with FFD registration are mostly visually similar to (a).

The first experiment seeks to verify the validity of the registration and evaluate the registration effect visually. The specific approach is to use the Philips DICOM Viewer to display the DWI, and the radiologist manually selects the pronounced deformation cases by observing all of the samples. After this step, the radiologist uses the “line function” of this software to measure the width or height of the prostate and then estimate the degree of deformation. The last step is to compare the visual effects of the first scheme with the third one and evaluate the degree of deformation compensation. The experimental results are shown in Figure 4.

As shown in Figure 4, the alignments of *b* = 500 s/mm^2^ DW image, *b* = 800 s/mm^2^ DW image, and *b* = 0 s/mm^2^ DW image can be compared in the “no processing” and “FFD” schemes (case 19). Figure 4 shows that DW image of *b* = 500 s/mm^2^ is similar to that of *b* = 0 s/mm^2^. Obviously, there is visual extrusion deformation in the prostate image of *b* = 800 s/mm^2^. However, this kind of image deformation can be compensated effectively in the FFD scheme. We also observe that few unrealistic artifacts are generated by FFD registration with different *b* value images, indicating that the adopted NCC is adequate.

### 4.3. Quantitative Evaluation of Motion and Deformation Compensation

The second experiment is dedicated to the quantization of alignment accuracy in the three schemes. Therefore, NMI of ROI is computed in compensation accuracy evaluation experiments. There are two ROIs per PCa patient, the lesion area located in the red circle and the general area located in the yellow circle, as shown in Figure 3. For each sample, two ROIs are defined with a diameter of 16 mm on the first *b* = 0 s/mm^2^ image in the “no processing” scheme. The same ROIs are used in the “affine” and “FFD” schemes. The ROI circle encompasses approximately 120 pixels. The ROI A (Figure 2) is manually positioned in the heterogeneous tumor region of the prostate. To compare the effect of the homogeneous region, the ROI B is selected in a common area as a reference for ROI A.

Table 1 reports the NMI values of each *b* value image to *b* = 0 s/mm^2^ image in all samples of the three schemes, which increased by 2.6% on average after affine transformation and 73.32% after nonrigid registration.

**Figure 5:**
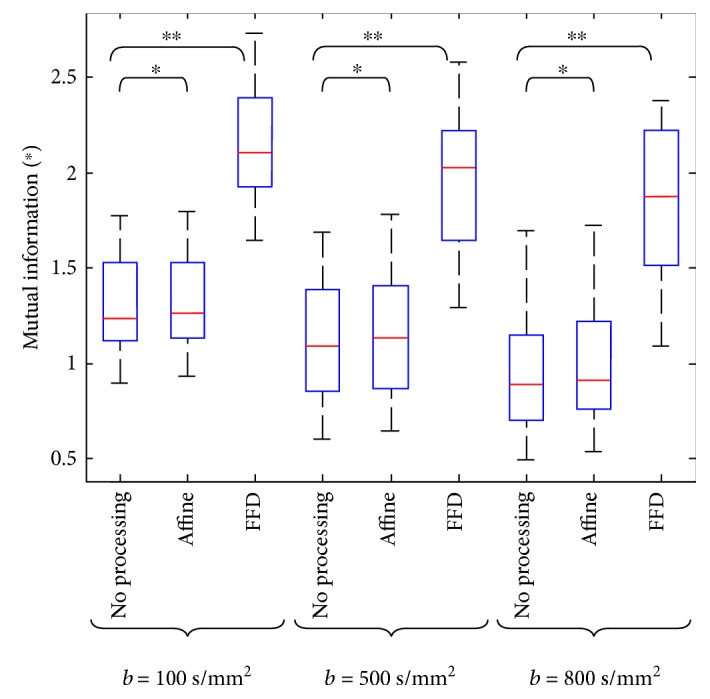
NMI with *b* = 100, 500, and 800 s/mm^2^ images and the *b* = 0 s/mm^2^ images. The highest NMI is obtained for the “FFD” scheme. Paired *t*-tests between the schemes: ^∗^*P* < 0.05, ^∗∗^*P* < 0.01.

Furthermore, to compare the compensation accuracy of the low *b* value (*b* = 100 s/mm^2^) image subset with the high *b* value (*b* = 500, 800 s/mm^2^) image subsets, in the three schemes, the paired *t*-test is implemented. The subsets' statistics results are shown in Figure 5.

In terms of NMI, Figure 5 shows that the aligned correlation in the “FFD” scheme is higher than that in the “no processing” and “affine” schemes with the respective mean NMI values of 1.3146, 1.3346, and 2.1696 for *b* = 100 s/mm^2^, 1.1284, 1.1627, and 1.9675 for *b* = 100 s/mm^2^, and 0.9482, 1.0133, and 1.8420 for *b* = 800 s/mm^2^. NMI tends to decrease gradually with increasing *b* values in the three schemes. The results show that NMI values are significantly different between the “no processing” and “FFD” schemes.

### 4.4. Quantitative Analysis of ADC Values

The third experiment seeks to compare the ADC values across the three schemes and evaluate the effect of the registration. The ADC median, homogeneity (using interquartile ranges (IQRs) [31]), mean level of diffusion (mean value) of ROIs, and the reproducibility (using the standard deviation (STD) of ADCs) across the three schemes are analyzed quantitatively with all samples. Paired *t*-tests are used to compare the distributions of the median ADC values, IQR, and mean values obtained in the three schemes.

**Figure 6:**
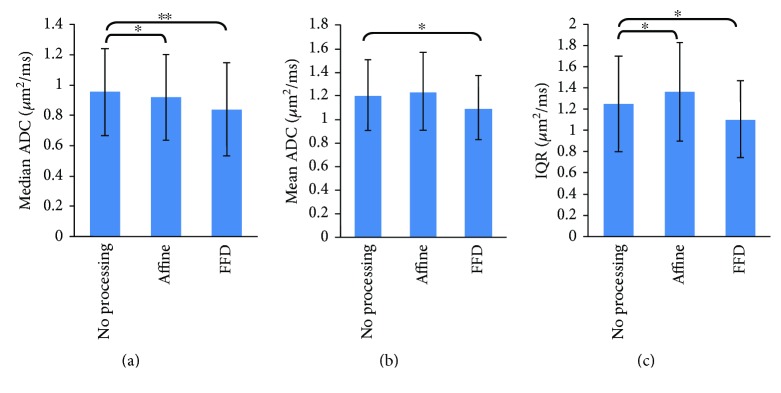
(a) Median ADCs, (b) mean ADCs, and (c) interquartile range (IQR) of ROI A are averaged over the 19 samples. The error bars represent the standard deviation of the measurements over 19 samples. Paired *t*-tests are used to compare the median ADC, mean ADC, and IQR datasets obtained in the “affine” and “FFD” schemes respect with the “no processing” scheme. ^∗^*P* < 0.05, ^∗∗^*P* < 0.01. Median ADCs, mean ADCs, and IQR obtained the lowest value in the “FFD” scheme.

The mean value, median value, and IQR computed from the ROI A of all datasets are reported in Table 2 and shown graphically in Figure 6. For a given dataset, the above quantitative parameters calculated from the two ROIs are quite similar. For all datasets, these parameters have a normal distribution. The main results corresponding to the average of the median ADCs, mean values, and IQRs of two ROIs are presented in Table 3.

The median ADCs of ROI A are 0.9523 in the “no processing” scheme, 0.9193 in the “affine” scheme, and 0.8385 in the “FFD” scheme. For ROI B, the respective median ADCs are 1.0707, 1.0335, and 0.9427. Statistics suggest that for ROI A, the median ADCs obtained in the “no processing” scheme are always higher compared with the “affine” (3.6%) and “FFD” (13.6%) schemes. The same trend occurs in ROI B. The respective mean ADCs of ROI A are 1.2070, 1.2388, and 1.1000 in the three schemes. For ROI B, the mean ADCs are 1.3543, 1.3900, and 1.2342, respectively. For both ROIs, the lowest mean ADCs are obtained in the “FFD” scheme, which indicates that the number of error values is partly reduced. Given a dataset, IQRs (characterizing the homogeneity of the ADCs) within both ROIs are lower with FFD registration than when “no processing” or “affine” transformation is applied to the images.  Table 4 shows that STDs are in general reduced in the “FFD” scheme, with respect to the “no processing” and “affine” schemes.

### 4.5. Visual Evaluation of ADC Map

**Figure 7:**
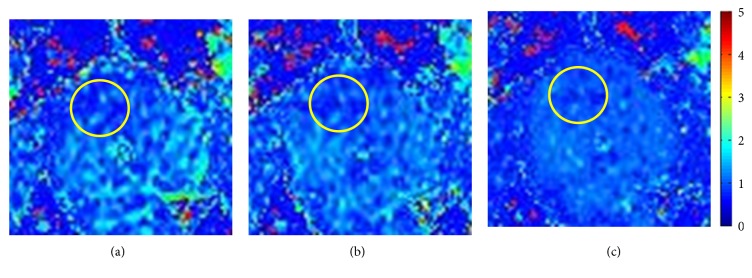
ADC maps of the 19th sample fitted with DWI. Color scales are given in *μ*m^2^/ms. (a) ADC map fitted with original DWI; (b) ADC map fitted with affine transformation DWI; (c) ADC map fitted with nonrigid registration DWI.

ADC maps are evaluated visually by radiologists in the three schemes. An example of ADC maps fitted with the 19th database is shown in Figure 7. Visual inspection of the ADC maps indicates that the “FFD” scheme improves the visual quality of the ADC maps with respect to the “no processing” scheme and the “affine” scheme: the suspected nidus areas are better visualized and the number of pixels for which the fitting fails to decrease. The ADC maps fitted with no processing sequence and affine transformation sequence visually appear to be similar.

The ADC map obtained by 7 *b* values ranging from 0 to 800 s/mm^2^ is shown in Figure 7(a). The affine and nonrigid registration results are shown in Figures 7(b) and 7(c), respectively. Alignment was assessed by two experienced radiologists who specialize in both T2WI and DWI. The local suspicious increase of intensity is visible in the high *b* value DW images. This region presents a diffusion-limited change, which was detected in the corresponding ADC map shown in Figure 7(a). However, the border of lesions is less clear in this ADC map fitted with the original images.  Figure 7(b) fitted with affine registration images displays the location and boundaries of the lesion more clearly. It also shows that the artifacts produced by deformation cannot be eliminated completely. In contrast, Figure 7(c) fitted with nonrigid registration images shows obvious diffusion-limited changes that are highly suspected of being PCa. It provides more accurate reference information for aspiration biopsy. This is also confirmed by the gold standard [8] for the diagnosis of prostate cancer. The lesion is confirmed to be PCa by TRUS biopsy with a Gleason score of 4 + 4 = 8.

The above evaluation and conclusion of the two radiologists illustrate that image registration is effective for addressing the deformation and motion of prostate DWI.

## 5. Discussion and Conclusion

In this study, we proposed a semiautomatic algorithm for the registration of prostate DWI to improve the ADC map. The influence of this image registration algorithm on ADC quantification was investigated in three schemes: no processing, with affine transformation, and with FFD registration of acquired DWI.

For the method of addressing the misalignment in DWI sequences, we apply affine transformation to eliminate interimage sliding motions and then use the nonrigid registration to correct intraimage deformations. This method with a global affine transformation is more time-efficient. NMI as the similarity measure is computed to elevate the registration result in both the “affine” and “FFD” schemes. The increase of NMI in the nonrigid registration scheme is distinct from that in the “affine” scheme, which indicates that the DWI is better aligned in the “FFD” scheme. It also shows that affine transformation is not sufficient for the alignment of prostate DWI and that the nonrigid registration with deformation compensation is compulsory. In addition, we assume that the dynamic SNR (Figure 2) with different *b* values in DWI sequence would challenge the validity of our algorithm. Therefore, low (*b* = 100 s/mm^2^) and high *b* value (*b* = 500, 800 s/mm^2^) subsets extracted from the acquired DWI are investigated. However, statistical results (Figure 4) show that our algorithm still performs well for different subsets. That is, our registration algorithm has good robustness.

The “no processing” and “affine” schemes result in the overestimation of the mean ADC values for ROI A of all datasets, by 9.7% and 12.6%, respectively, and the IQRs are overestimated by 13.1% and 23.1%, respectively, compared with FFD scheme. The median ADC in the “no processing” scheme is overestimated by 3.59% and 13.6%, respectively, compared with that in the “affine” scheme and “FFD” scheme. Variability measure (STD) is generally improved with FFD registration. Similar trends also appear in ROI B for median and mean ADCs. However, IQR values of ROI B have no significant difference with or without registration. This indicates that the registration algorithm can make the lesion boundaries clearer in the inhomogeneous region (ROI A). These effects are visually confirmed in Figure 7(). These results indicate that FFD registration significantly improves the visual quality of ADC maps. It also suggests that the effect of registration on the ADC maps is mainly determined by the deformation compensation of the prostate. This compensation of deformation is influenced definitively by the FFD grid resolution: a higher resolution would obtain a better effect. However, higher resolution is time-consuming, so the highest resolution in this paper is limited to within 90 × 90. The experimental results indicate that the algorithm with this resolution is capable to be sufficiently accurate.

The radiologists observed and analyzed the malignant regions while reviewing these T2-weighted images and DWI and ADC maps. They found that the ADC maps are valid in the “affine” and “FFD” schemes. The ADC maps of the “FFD” scheme provide the most details for the clinical staging of PCa. Therefore, the ADC maps of the “FFD” scheme are accepted by radiologists and correspond with the clinical diagnosis. The registration algorithm can eliminate the drift and deformation of the acquired DWI. This indicates that our method in this study is efficient.

In conclusion, this study shows that image registration can correct the misalignment of prostate DWI and improve ADC maps. The clinicians believe that the ADC maps obtained from the registered DWI are more effective for the detection, diagnosis, and staging of PCa. For future work, it could be interesting to compare this method with the use of a nonrigid 3-D transformation model, correcting for both intraslice and interslice motions.

## Figures and Tables

**Table 1:** NMI values of ROI A.

	*b* values				
	50	100	200	500	800
No processing	1.3883 ± 0.3239	1.3649 ± 0.4168	1.1993 ± 0.3255	1.0995 ± 0.3354	0.9230 ± 0.3511
Affine	1.4051 ± 0.3136	1.3285 ± 0.2773	1.2353 ± 0.3226	1.1405 ± 0.3375	0.9945 ± 0.3179
FFD	2.2686 ± 0.3576	2.1717 ± 0.3192	2.0518 ± 0.3595	1.9412 ± 0.3756	1.8141 ± 0.4151

**Table 2:** Mean value, median value, and interquartile ranges (IQRs) of ADC values of ROI A.

		No processing	Affine	Nonrigid			No processing	Affine	Nonrigid
Case 1	ADC median	1.0223	1.0210	1.0254	Case 11	IQR	1.6132	1.6954	1.5387
	Mean	1.0783	1.0165	1.0312		ADC median	0.6351	0.6318	0.5758
	IQR	1.0122	1.0166	1.0291		Mean	1.0238	1.4928	1.1099
Case 2	ADC median	0.8718	0.8643	0.8333	Case 12	IQR	1.0282	1.5977	1.1027
	Mean	0.95283	0.95735	1.0243		ADC median	0.6161	0.5463	0.4804
	IQR	0.94842	0.95098	1.0026		Mean	0.9565	1.1463	0.7726
Case 3	ADC median	0.7015	0.7474	0.7198	Case 13	IQR	0.9780	1.1532	0.8041
	Mean	0.9460	1.0262	1.0247		ADC median	0.9387	0.9678	0.8982
	IQR	1.0927	1.3608	0.9896		Mean	1.2657	1.3359	1.3019
Case 4	ADC median	1.2350	0.9852	0.9813	Case 14	IQR	1.1897	1.2429	1.2441
	Mean	1.1309	0.9509	0.9042		ADC median	1.0832	1.1090	0.4436
	IQR	1.3482	1.2789	1.2425		Mean	1.7366	1.7929	0.9207
Case 5	ADC median	1.0299	1.0054	0.9795	Case 15	IQR	2.0324	2.1214	0.9971
	Mean	1.1452	1.0806	1.0023		ADC median	1.2260	1.1649	1.0726
	IQR	0.8864	0.8529	0.7936		Mean	1.9196	1.9111	1.6321
Case 6	ADC median	1.0833	0.9710	0.9115	Case 16	IQR	2.5168	2.4777	2.1027
	Mean	1.0994	1.0265	1.1976		ADC median	1.0859	1.0600	1.0531
	IQR	0.8550	0.89675	0.81655		Mean	1.2464	1.3655	1.2247
Case 7	ADC median	0.8444	0.8440	0.8330	Case 17	IQR	0.9431	0.9959	0.7185
	Mean	1.1060	1.1296	1.1780		ADC median	0.8975	0.8090	0.6590
	IQR	1.5310	1.5939	1.5410		Mean	1.3103	1.6779	1.0683
Case 8	ADC median	1.7388	1.7637	1.7112	Case 18	IQR	1.2911	1.9409	0.9682
	Mean	1.6751	1.6630	1.7845		ADC median	0.9277	0.9185	0.8545
	IQR	1.5764	1.5617	1.5488		Mean	1.4199	0.9185	1.2287
Case 9	ADC median	0.7816	0.7363	0.6901	Case 19	IQR	1.2304	1.2366	1.0727
	Mean	0.7934	0.7469	0.6692		ADC median	0.3949	0.4006	0.2846
	IQR	0.6996	0.6860	0.6778		Mean	0.9149	1.0515	0.8216
Case 10	ADC median	0.9801	0.9200	0.9302		IQR	0.9547	1.1746	0.7876
	Mean	1.2127	1.2478	1.2043					

All values are given in *μ*m^2^/ms.

**Table 3:** Average of the median ADCs, mean values, and IQRs.

		No processing	Affine	Nonrigid
(a)				
Mean ± STD	ADC median	0.9523 ± 0.2863	0.9193 ± 0.2820	0.8385 ± 0.3057
	Mean	1.2070 ± 0.2983	1.2388 ± 0.3290	1.1000 ± 0.2708
	IQR	1.2488 ± 0.4483	1.3597 ± 0.4647	1.1041 ± 0.3631

(b)				
Mean ± STD	ADC median	1.0707 ± 0.1632	1.0335 ± 0.3581	0.9427 ± 0.3527
	Mean	1.3543 ± 0.1650	1.3900 ± 0.3165	1.2342 ± 0.1742
	IQR	1.4812 ± 0.4368	1.5256 ± 0.3238	1.3388 ± 0.3554

All values are given in *μ*m^2^/ms. The average lines contain mean and standard deviation values calculated with all of 19 datasets. (a) ROI A, (b) ROI B.

**Table 4:** STD of ADCs of ROI A.

	Case1	Case2	Case3	Case 4	Case 5	Case 6	Case 7	Case 8	Case 9	Case10
No processing	0.6649	0.6205	0.7803	0.7346	0.6961	0.5821	0.8673	0.9296	0.4608	0.8952
Affine	0.6725	0.6179	0.8598	0.6989	0.7861	0.5980	0.8955	0.9325	0.4781	0.9260
FFD	0.5159	0.4314	0.4464	0.5537	0.5358	0.4364	0.6457	0.8458	0.3066	0.8344

	Case11	Case12	Case13	Case14	Case15	Case16	Case17	Case18	Case19	

No processing	1.1471	1.1374	1.1949	1.8482	1.8605	0.9684	1.3349	1.5329	1.3889	
Affine	1.5842	1.3482	1.2936	1.8832	1.8377	1.0537	1.5392	1.5870	1.5918	
FFD	1.0058	1.1089	1.0955	1.3184	1.6354	0.9447	1.2388	1.3291	1.1266	

All values are given in *μ*m^2^/ms.
